# Cinnamaldehyde Loaded Poly(lactide-co-glycolide) (PLGA) Microparticles for Antifungal Delivery Application against Resistant *Candida albicans* and *Candida glabrata*

**DOI:** 10.3390/plants12132437

**Published:** 2023-06-24

**Authors:** Silvia Rizzo, Maura Di Vito, Elena Mazzinelli, Ilaria Favuzzi, Riccardo Torelli, Margherita Cacaci, Alessandro Arcovito, Maurizio Sanguinetti, Stefania Garzoli, Giuseppina Nocca, Francesca Bugli

**Affiliations:** 1 Dipartimento di Scienze Biotecnologiche di Base, Cliniche Intensivologiche e Perioperatorie, Università Cattolica del Sacro Cuore, 00168 Rome, Italy; silvia.rizzo@unicatt.it (S.R.); maura.divito@unicatt.it (M.D.V.); elena.mazzinelli@unicatt.it (E.M.); ilaria.favuzzi@unicatt.it (I.F.); margherita.cacaci@unicatt.it (M.C.); alessandro.arcovito@unicatt.it (A.A.); giuseppina.nocca@unicatt.it (G.N.); francesca.bugli@unicatt.it (F.B.); 2 Dipartimento di Scienze di Laboratorio e Infettivologiche, Fondazione Policlinico Universitario A. Gemelli IRCCS, Largo A. Gemelli 8, 00168 Rome, Italy; riccardo.torelli@policlinicogemelli.it; 3 Fondazione Policlinico Universitario A. Gemelli, IRCCS, Largo A. Gemelli 1, 00168 Rome, Italy; 4 Dipartimento di Chimica e Tecnologie del Farmaco, Università di Roma Sapienza, Piazzale Aldo Moro 5, 00185 Rome, Italy

**Keywords:** cinnamaldehyde, PLGA, microparticles, SPME-GC-MS analysis, *C. albicans*, *C. glabrata*

## Abstract

Researchers have explored natural products to combat the antibiotic resistance of various microorganisms. Cinnamaldehyde (CIN), a major component of cinnamon essential oil (CC-EO), has been found to effectively inhibit the growth of bacteria, fungi, and mildew, as well as their production of toxins. Therefore, this study aimed to create a delivery system for CIN using PLGA microparticles (CIN-MPs), and to compare the antifungal activity of the carried and free CIN, particularly against antibiotic-resistant strains of *Candida* spp. The first part of the study focused on synthesizing and characterizing the PLGA MPs, which had no toxic effects in vivo and produced results in line with the existing literature. The subsequent experiments analyzed the antifungal effects of MPs-CIN on *Candida albicans* and *Candida glabrata*, both resistant (R) and sensitive (S) strains and compared its efficacy with the conventional addition of free CIN to the culture medium. The results indicated that conveyed CIN increased the antifungal effects of the product, particularly towards *C. albicans* R. The slow and prolonged release of CIN from the PLGA MPs ensured a constant and uniform concentration of the active principle within the cells.

## 1. Introduction

Candidiasis is a class of fungal infections caused by yeast of the genus *Candida*. They are in fourth place as infectious agents causing nosocomial diseases and septicemia and in first place for mortality rate associated to them [1]. From a clinical point of view, the more significant among *Candida* species is *C. albicans*, isolated in approximately 60% of patients with invasive candidiasis [2], *C. albicans* is a polymorphic saprophytic fungus and a common microbiota component in around 50% of the population. It could be found in mucosal surfaces of gastrointestinal (from oral cavity to anus), respiratory and genitourinary tracts [3]. It is generally a harmless commensal fungus, but it causes chronic mucocutaneous and systemic infections in immune-compromised individuals [4], with a mortality rate of among 40% [5]. In these patients, *C. albicans* gradually occupies “niches” microbially imbalanced due to excessive hospital hygienic procedures and antibiotic therapies [6]. So, *C. albicans* is an extremely plastic species, able to adapt to host conditions and to be harmless in healthy people and pathogenetic in clinically compromised patients [7].

Although *C. albicans* is the main cause of human candidiasis, however, the number of infections caused by Non-*C. albicans Candida* (NCAC) species has significantly increased over the last years [8,9]. *C. glabrata*, *C. parapsilosis* and *C. tropicalis* are the most common candidiasis cause species, after *C. albicans. C. glabrata* is a non-polymorphic saprophytic fungus, generally non pathogenetic, present in the intestinal eubiotic microbiota of healthy people. However, the increased use of immunosuppressive and antimicrobial therapies caused an increase of mucosal and systemic *C. glabrata* infection [10].

Currently, four class of traditional antifungal drugs are used by clinicians and veterinaries for systemic treatment of fungal infections. Antifungal classes are polyenes, azoles, echinocandins and flucytosine. In recent decades, the frequent and prophylactic use of antifungal drugs has led to the development of strong resistance in many *Candida* species. This represents a significant clinic threat that can cause new epidemics and millions of deaths, so, in recent years, a lot of natural origin molecules are studied as potential antimicrobial agents. Plant extracts, essential oils and their derivates are active against a wide variety of microorganisms [11]. Considerable interest is given to the study of cinnamaldehyde (CIN), one of the main active components of cinnamon essential oil. It is in *Cinnamomun* genus trees bark, *zeylanicum*, (or *verum*), *canfora* and *cassia* species [12]. It has been shown that CIN effectively inhibits bacteria, fungus and mildew growth and their production of toxins [13]. Experimental evidence suggests that CIN shows antifungal activity by inhibiting cell wall biosynthesis, cellular membrane function and to specific enzyme [14]. CIN is a safe natural well-tolerated molecule by human and animals, approved by the Food and Drug Administration (FDA) and the European Council with daily administration within 1.25 mg/kg depend on body weight. 

Antifungal drugs administration in free form has several disadvantages such as reduced selectivity for pathogenic microorganisms and the risk of compound degradation before reaching the target site [15]. Moreover, because of the acquired resistance by many microorganisms, the administration of a high dose medication is required to have a therapeutic effect. 

This leads to a higher risk for systemic toxicity and side effects such as allergic reaction, hepatoxicity, nephrotoxicity and damage to the healthy microbiota [16]. The use of drug delivery systems can overcome these disadvantages. Finally, drug delivery systems are advantageous for active compounds with low solubility and stability. In last decades, more and more biodegradable synthetic polymers have been used as vehicles for therapeutic drug release devices, such as polylactic-co-glycolic acid, or PLGA, approved by European Medicines Agency and FDA. Thanks to its biocompatibility, adequate biodegradation kinetics and low toxicity, it represents an ideal polymer for nanomedicine applications [17].

The present work was focused on the preparation and characterization of the CIN, from *Cimmanomum cassia* essential oil (CC-EO), delivery system based on PLGA microparticles (MPs). Scanning Electron Microscopy (SEM) imaging, CIN entrapment efficiency and in vitro release studies of CIN demonstrated an excellent development of the delivery system. The antifungal activity of MPs-carried CIN in comparison with CIN alone was analysed by Minimal Fungicidal Concentration (MFC90) measurement and cell membrane integrity evaluation. Particular attention was paid to the antifungal activity against drug-resistant candida isolates. Further, safety of compounds was assessed in *Galleria mellonella* in vivo model. Taken together, our data reveal high antifungal efficacy of CIN and CIN-MPs which overcomes resistance mechanisms towards common antifungal drugs.

## 2. Results

### 2.1. SPME-GC-MS Analyses 

**Figure 1:**
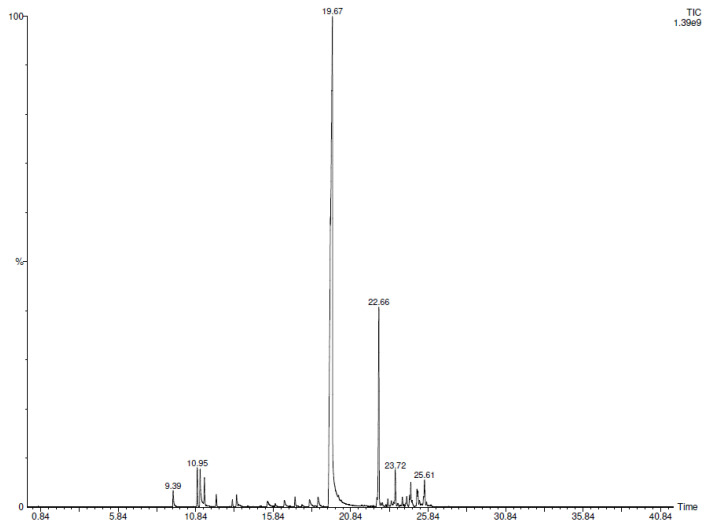
GC-FID Chromatogram of CC-EO.

SPME-GC-MS (Solid Phase Microextraction-Gas Chromatography-Mass Spectrometry) analysis allowed the identification of twenty-eight volatile components emitted by CC-EO (Table 1). The major compound was trans-CIN (70.4%) followed by α-copaene (9.8%) and a series of minor compounds with a percentage value ranging from 0.1% to 2.7%. The chromatogram is also reported (Figure 1).

### 2.2. Preparation and Characterization Analysis of PLGA CIN-MPs

**Figure 2:**
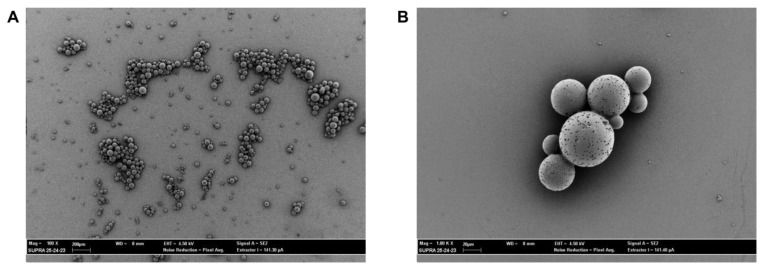
Representative SEM images of empty-MPs, scale bar 200 µm (**A**; Mag = 100 X) and 20 µm (**B**; Mag = 1.00 KX).

The morphology of MPs was studied by scanning electron microscopy (SEM). Representative images of empty-MPs are shown in Figure 2, where the observed morphology appears to be spheroidal, with a smooth surface, and with heterogeneous dimensions with diameters greater than 1 µm. CIN loading into MPs do not alter significantly their morphology, as can be deduced from Figure 2. 

### 2.3. Cinnamaldehyde Entrapment Efficiency and In Vitro Release Studies

The total amount of CIN loaded in 1 mg MPs (loading) was 120 µg (so, 8.4 mg of CIN in 70 mg of loaded MPs obtained). Since 40% *v*/*w* of CIN was used in the preparation, the entrapment efficiency is 21%. 

CIN release profiles were evaluated in vitro as a function of time (at 1, 4 and 24 h) in three different conditions (in ethanol, in Sensititre™ YeastOne Broth and in Sensititre™ YeastOne Broth in presence of *Candida* spp.). The temperature used during the test (37 °C) was chosen to resemble ideal microbial growth conditions.

**Figure 3:**
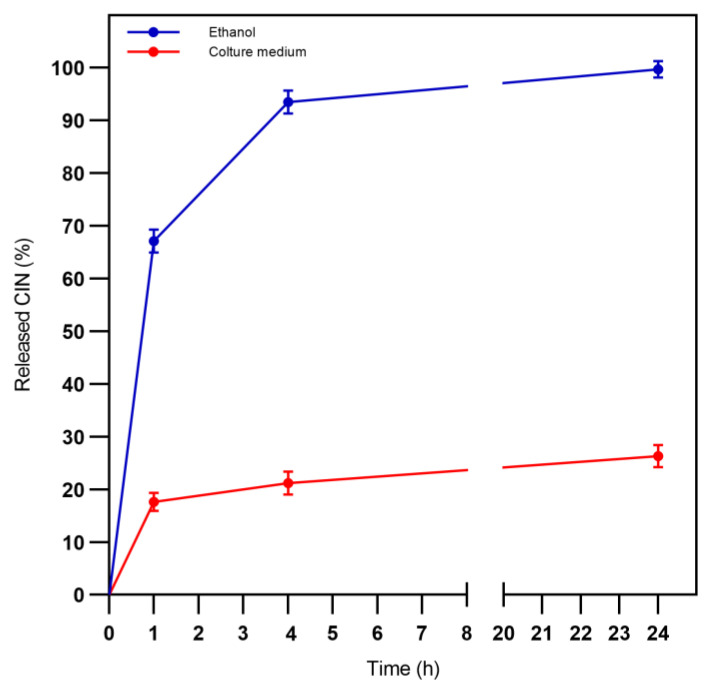
In vitro drug release profiles of CIN-MPs in ethanol and culture medium. Results were represented as mean ± St. Dev. Analyses were performed in triplicate.

As shown in Table 2 and Figure 3, in ethanol, CIN-MPs presented the highest initial burst release, reaching almost 67% of the CIN’s amount originally present in the sample and about 100% was released after 24 h. In Sensititre™ YeastOne Broth after 24 h, CIN-MPs released 26,30% of the CIN. The difference in CIN release profiles in ethanol and in Sensititre™ YeastOne Broth is statistically significant (*p* value < 0.0001).

Finally, as shown in Table 3 CIN release was almost total in presence of *C. albicans* or *C. glabrata* on a growth area of 1.90 cm^2^, in 24 h, for all three concentrations analyzed. The difference in release percentage between 600 µg/mL and 300 µg/mL showed slightly significant differences (*p* value = 0.0031) while there was no significant differences be-tween 300 µg/mL and 150 µg/mL. In wells with growth area equal to 0.31 cm^2^, the release decreased as the concentration increased. This phenomenon was due to the aggregation of the MPs in a restricted area and the reduced surface of the same in contact with the ground that promoted the release. Moreover, the release in the presence of *Candida* increased as the gradual release of CIN by the MPs resulted in the gradual uptake of the molecule by the microorganisms. The difference in CIN release percentage in growth area equal to 0.31 cm^2^, at the three concentrations analyzed was statistically significant (*p* value < 0.0001).

**Figure 4:**
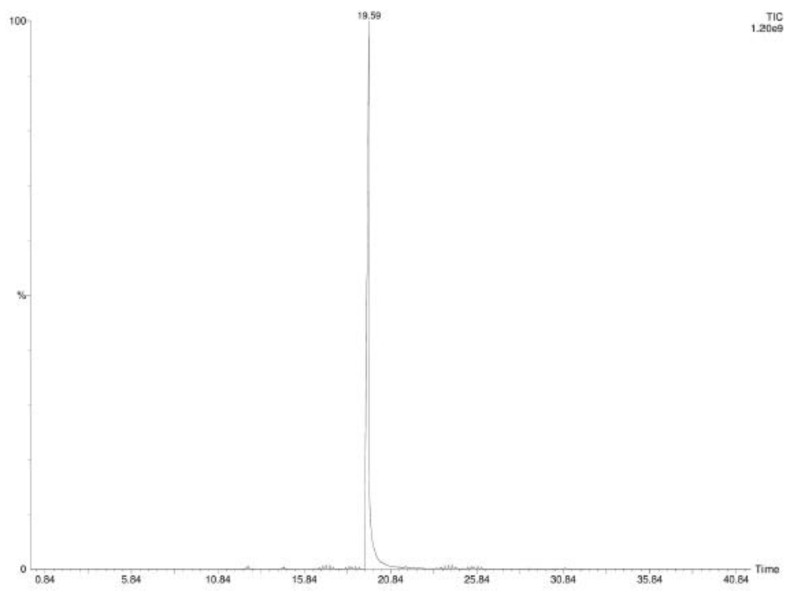
GC-FID Chromatogram of CIN-MPs.

Headspace analysis of the vehiculate CIN confirmed the absence of other metabolites. The chromatogram is also reported (Figure 4).

### 2.4. In Vivo Toxicity in Galleria Mellonella Model

**Figure 5:**
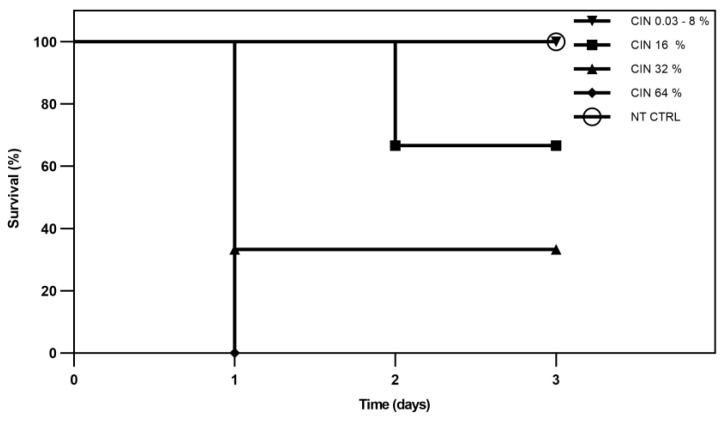
In vivo toxicity of non vehiculated CIN on *G. mellonella* (*p* value < 0.0001).

The toxicity tests results of non vehiculated CIN on *G. mellonella* model showed total survival in the concentration range 300–80,000 μg/mL and in the untreated group (NT CTRL) after 72 h. Larvae treated with CIN showed survival rate equal to 70% after 72 h at 160,000 μg/mL, equal to 30% at 320,000 μg/mL and equal to 0% after 24 h at 640,000 μg/mL (Figure 5). All these conditions showed significant differences vs. NT CTRL (*p* value < 0.0001).

### 2.5. Antifungal Activity

The MFC90 values and their standard deviations (St. Dev.) of vehiculated and non-vehiculated CIN and CC-EO against *C. albicans* and *C. glabrata* strains were summarized in Table 4. The evidenced MFC90 values of CIN vehiculated, non-vehiculated and CC-EO were among respectively 400 µg/mL and 500 µg/mL for all selected Candida species, while only *C. albicans* azole-resistant showed a lower MFC90 value (MFC90 200 µM). The efficiency control, performed with fluconazole, showed MIC (minimum inhibitory concentration) values equal to 0.25 μg/mL for sensible strain of *C. albicans*, equal to 16 μg/mL for sensible strain of *C. glabrata* and >128 μg/mL for resistant strain of *C. albicans* and *C. glabrata*. No statistical differences were showed for different treatments and among the distinct strains.

### 2.6. SEM Evaluation

**Figure 6:**
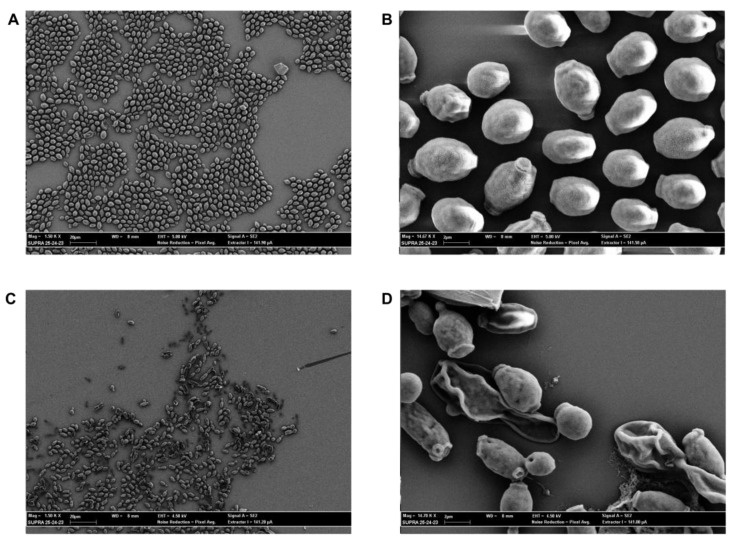
Representative SEM images of untreated (**A**, Mag = 1.50 KX; **B**, Mag= 14.67 KX) and treated with MFC90 values of CIN-MPs (**C**, Mag = 1.50 KX; **D**, Mag= 14.67 KX) *C. albicans* resistant.

**Figure 7:**
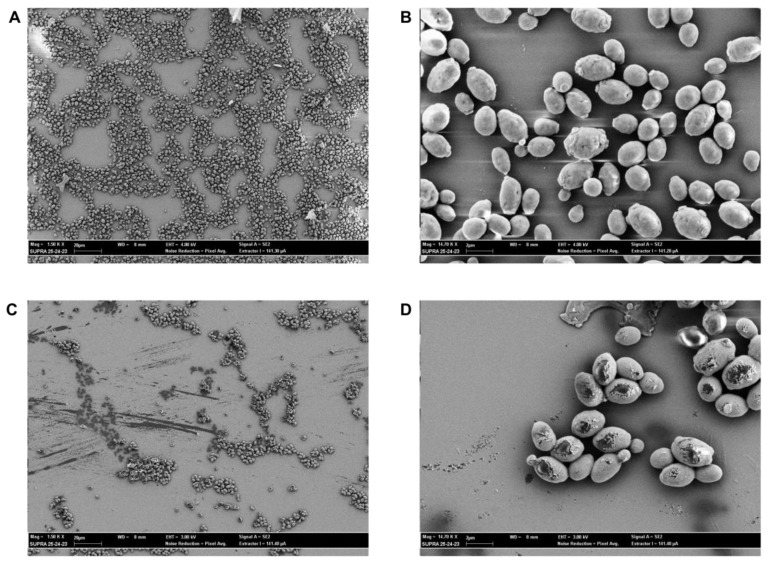
Representative SEM images of untreated (**A**, Mag = 1.50 KX; **B**, Mag= 14.67 KX) and treated with MFC90 values of CIN-MPs (**C**, Mag = 1.50 KX; **D**, Mag= 14.67 KX) *C. glabrata*.

Antifungal activity of CIN and CIN-MPs at MFC and sub-MFC concentrations was supported by SEM images. The images showed that CIN and CIN-MPs, at MFC90 values, were able to damage yeast cells whose shape appears atypical with clear signs of suffering and hyphaea shape appears crumple. Untreated cells and cells treated with sub-MFC concentration values, showed round and homogeneous shape and hyphae appear turgid and tubular (Figure 6 and Figure 7).

### 2.7. Effect of CIN and CIN-MPs on Cell Membrane Integrity

The nucleic acid content (OD260nm) of fungal suspension of treated group (with CIN and CIN-MPs at MFC 90 concentrations), increased significantly compared to untreated control group (Table 5). CIN and CIN-MPs treatments showed a statistically significant activity (*p* value < 0.0001) compared with the untreated controls for all the three strains analyzed.

## 3. Discussion

Plants represent an extraordinary resource for the isolation of compounds with antimicrobial activity. Cinnamaldehyde used in this study derived from *Cinnamomum cassia* (CC) Essential Oil (EO). Table 1 shows the chemical volatile composition of CC EO and CIN-MPs obtained by SPME-GC-MS analysis. The content of CIN in CC EO resulted equal to 70.4% as the main component, while the only CIN phytocompound encapsulated in microparticles, reaches 100%. The MFC values obtained with EO are comparable to those obtained with CIN alone. This demonstrates, as known by previous works, that the main component of this phytocomplex is responsible for antifungal properties [18,19,20]. EOs and their active components are generally characterized by some drawbacks such as high volatility, chemical instability, and low solubility in water, hence their limited use in medical therapy [21,22]. To overcome these limitations, microparticles are extensively applied as carriers for drug delivery, because they can prevent the degradation of the drug, can target the active compounds to the specific tissue, can reduce the side effects, etc. [23]. A lot of polymers are used as vectors for drug delivery, among these, PLGA, characterized by good biocompatibility, is widely applied for this purpose. In this study, the single emulsion technique, was used to incapsulate CIN inside PLGA microparticles. In fact, this technique, is particularly used to incapsulate hydrophobic compounds.

The encapsulation of CIN, within micro or nano vectors, has been performed in other works and with different aims [24,25,26]: antimicrobial activity in the oral cavity, in bone, and in food conservation. On the base of the literature data, we decided to extend our studies also on antibiotic-resistant Candida strains. 

For this purpose, we characterized the microparticles produced evaluating load, encapsulation efficiency, and release of the active ingredient in different media. In terms of encapsulation efficiency, the obtained results are consistent with the literature considering the ratios between the different compounds used in the single emulsion reaction [27].

Regarding the release curves of the CIN from the microparticles, it is interesting to observe how, in ethanol, the release has the usual initial burst of the particles in PLGA [27], in these conditions, in fact, the medium used is a solvent for CIN. When the test is repeated in a *Candida* culture medium, i.e., in a non-solvent medium, the release becomes much more gradual and reaches, after 24 h, an amount equal to ¼ of the total encapsulated CIN. The most interesting results are observed when the CIN release is carried out in the culture medium in the presence of *Candida*, in this case, in 24 h the release is practically complete, indicating that the active principle penetrates inside the microorganism favouring the “emptying” of the microparticles gradually. This particular effect can be deduced from the higher antifungal efficacy observed when CIN is administered within the microparticle compared to the free form: it is conceivable that the vehiculation maintains the concentration of CIN at fungicidal concentration longer than the non-vehicle form, with a consequent increase in the antimicrobial effect. This is particularly evident with *C. albicans* R. As shown in Table 4, efficiency of CIN and CIN-MPs, among the distinct strains, doesn’t show significant statistical differences, unlike the fluconazole efficiency.

Furthermore, SEM images (Figure 6 and Figure 7) of the two resistant isolates of *C. albicans* and *C. glabrata* treated with free and microencapsulated CIN show a clear morphological alteration when compared with the images of the untreated cells. Cells integrity appears seriously compromised, with evident pores on the fungal wall or with the presence of completely lysed cells (Figure 6C,D and Figure 7C,D). These results are in line with what was obtained on the release of nucleic acids following treatment of *Candida* spp. with CIN and CIN-MPs at the MFC concentration. The evaluation of the release of biological macromolecules from the cytoplasm to the outside of the cell is widely used for the evaluation of the integrity of the microbial cell wall and cytoplasmic membranes [28]. Finally, the evaluation of the total absence of toxicity in the *Galleria mellonella* model, at a concentration 1000 times higher than that of MFC, makes this micro-composite suitable for future applications in clinical therapy.

## 4. Materials and Methods

### 4.1. Essential Oil and Active Component

The essential oil *Cinnamomum cassia* (CC-EO by Pranarôm International, Avenue des Arti-sans, Ghislenghien, Belgium) and cinnamaldehyde (CIN by Ventos, Barcelona, Spain, batch L4412580), its main component, were used for the study.

### 4.2. Solid Phase Microextraction (SPME) Sampling

To describe the volatile chemical profile of CC-EO and CIN-MPs, a SPME fiber was used. Approximately 0.5 mL of each sample was individually placed into a 7 mL glass vial with polytetrafluoroethylene (PTFE)-coated silicone septum. Before the sampling, a thermostatic bath with constant magnetic stirring was used for 15 min to reach thermal equilibrium. The extraction of volatile compounds was performed by using a SPME device from Supelco (Bellefonte, PA, USA) equipped with a 1 cm fiber coated with 50/30 μm DVB/CAR/PDMS (divinylbenzene/carboxen/polydimethylsiloxane). The fiber was first conditioned at 270 °C for 30 min and then it was inserted into the vials and exposed to the headspace for 5 min at 50 °C. Lastly, the SPME fiber was inserted to the GC injector port set to 250 °C in splitless mode for the desorption.

### 4.3. GC-MS Analyses of CC-EO and CIN-MPs

The headspace analysis by SPME of samples were carried out using a Clarus 500 model Perkin Elmer (Waltham, MA, USA) gas chromatograph coupled with a mass spectrometer and equipped with a FID (flame ionization detector) [19,29]. The capillary column used for the separation of compounds, was a Varian Factor Four VF-1. The operative chromatographic and spectrometric conditions were as follows: the oven GC temperature program was: isothermal at 60 °C for 5 min, then ramped to 220 °C at a rate of 6 °C min^−1^, and finally isothermal at 220 °C for 20 min. The carrier gas was He at flow rate of 1.0 mL min−1 in constant mode. The mass spectra were obtained in the electron impact mode (EI), at 70 eV, in scan mode in the range 35–450 *m*/*z*. For the identification of compounds, the matching between their mass spectra with those stored in the Nist 02 mass spectra library database, was performed. Further, the linear retention indices (LRIs), were calculated using a series of alkane standards (C_8_–C_25_ *n*-alkanes) and compared with those available in the literature. Relative amounts of compounds, expressed as percentage, were calculated in relation to the total area of the chromatogram by normalizing the peak area without the use of an internal standard and any factor correction. All analyses were carried out in triplicate.

### 4.4. Synthesis of Microparticles PLGA-CIN Microparticles and Production Efficiency

The MPs were prepared by a single emulsion solvent evaporation technique. Briefly, for organic phase preparation 25 mg of poly(lactide-co-glycolide) (PLGA) (L-lactide:glycolide 75:25, molecular weight 66–107 kDa, Sigma-Aldrich) and 40% *v*/*w* of CIN (density of 1.05 g/cm^3^) were dissolved in 1 mL of dichloromethane (DCM). The organic phase was emulsified to an aqueous phase (PVA 0.3% *w*/*v* in 10 mL of distilled water) through sonication in a probe sonicator (Vibra, Bioblock Scientific, France) for 5 min with 70% voltage efficiency at 25 °C. To allow organic phase evaporation, the emulsion was stirred (700 rpm for 3 h) at room temperature. Once synthesized, PLGA-CIN MPs were washed twice in distilled water using an ultracentrifuge (Avanti J-25 Beckman, California) (16,000 rpm for 30 min at 10 °C). Finally, the MPs resuspended in distilled water were kept at −20 °C overnight and freeze-dried at −80 °C under vacuum for 48h. Dried MPs were collected and stored at room temperature. The production efficiency was calculated according to Equation (1).

(1) $$\mathrm{Production}\;\mathrm{efficency}\;\left(\%\right)=\frac{\mathrm{amount}\;\mathrm{of}\;\mathrm{dried}\;\mathrm{MPs}}{\mathrm{initial}\;\mathrm{PLGA}\;\mathrm{amount}}\times 100\;$$

### 4.5. Microparticles Size and Morphology

MPs size and the morphology were examined by scanning electron microscopy (SEM, Supra 25′, Zeiss, Germany). A droplet of the aqueous phase containing MPs was placed on aluminium stub covered with a conductive carbon disk, it was dried and metallized with a 40 nm thick gold film using a sputter coater (High Resolution Sputter Coater AGB7234, UK) (19.30 g/cm^3^ and 40 mA/s) before analysis by SEM. Observations were performed at 100–1000 X. 

### 4.6. Microparticles Entrapment Efficiency (EE) and Cinnamaldehyde Release Studies

The entrapment efficiency of CIN in the PLGA MPs was measured by reverse- phase (RP)-HPLC. A sample of 1 mg lyophilized MPs was dissolved in 1 mL of acetonitrile and water (95:5) and was filtered, using a 0.22 μm filter, before HPLC analysis. A C-18 reverse-phase column (4.6 mm × 250 mm, 5 μm) was used. The mobile phase consisted of acetonitrile: water: acetic acid in a ratio of 50: 50: 0.01, with a flow isocratic rate of 300 μL/min and a total run time of 40 min per sample. Analysis wavelength was 295 nm. 

MPs loading was calculated according to Equation (2):

(2) loading=CIN∈1mg di MPs×mg lyophilized MPs 

Then, the entrapment efficiency (EE) was calculated according to Equation (3):

(3) $$\mathrm{EE}\;\left(\%\right)=\frac{\mathrm{loading}}{\mathrm{initial}\;\mathrm{CIN}\;\mathrm{amount}}\times 100$$

All experiments were performed in triplicate.

For in vitro release studies 2 mg of lyophilized CIN-MPs were dispersed in 2 mL of ethanol under magnetic stirring (700 rpm). At fixed time intervals (1, 4 and 24 h), the sample was centrifuged at 11,000 rpm for 15 min and 1 mL of the supernatant was withdrawn and replaced with 1 mL of fresh ethanol. The supernatant was filtered with a 0.22 μm filter and the released CIN was measured by reverse- phase (RP)-HPLC at the same conditions previously described. The release study in fungal cell culture medium (Sensititre YeastOne Broth, USA), both in presence and in absence of *Candida* spp., was performed with the same procedure utilized in ethanol. 

### 4.7. In Vivo Toxicity Study

The toxicity of CIN and CIN-MPs was evaluated in an in vivo model of *G. mellonella* placed at 33 °C in an aerobic incubator. Larvae with colour changes in the body and larvae outside the body weight of 0.2–0.3 g were excluded. Larvae were treated with 300, 600, 1250, 2500, 5000, 10,000, 20,000, 40,000, 80,000, 160,000, 320,000 and 640,000 µg/mL (or 0.03–8%, 16%, 32% and 64% *v*/*v*) of CIN free and 600, 300 and 150 µg/mL of CIN-MPs by injecting 10 μL of solution (in physiological solution) into the haemocoel through the last left pro-leg of ten larvae using a 0.5 mL syringe. The injection area was decontaminated with 70% ethanol prior to administration. The larvae were observed every after 24 h, 48 h and 72 h. 

### 4.8. Clinical Strains of C. albicans and C. glabrata

Two clinical strains of *C. albicans* and two of *C. glabrata* (for each species one azole-sensitive and one azole-resistant) isolated from positive blood cultures and provided by the UOC of Microbiology of Policlinico Universitario A. Gemelli of Rome, Italy, were used. Sensititre™ YeastOne Broth (Thermo Fisher, Waltham, MA, USA) and Sabouraud Dextrose Agar (Oxoid, Wade Road, Basingstoke, Hants, UK) were used to growth strains at 37 °C for 24 h.

### 4.9. Antifungal Activity 

In order to determine the antifungal efficacy of the CIN, CIN-MPs, and EO minimum inhibitory concentration (MIC) and minimum fungicide concentration (MFC) were determined using a broth microdilution assay according to the criteria proposed by EUCAST (Subcommittee on Antifungal Susceptibility Testing, AFST) of the ESCMID (European Committee for Antimicrobial Susceptibility Testing EUCAST, 2008). The broth microdilution test was performed on a flat bottom 24-well microplates by adding 100 μL of a fungal suspension equal to 5 × 10^5^ CFU/mL to a final volume of 200 μL. Scalar dilution for CIN, CIN-MPs and EO (range concentration from 1250 µg/mL to 75 µg/mL) were prepared in Sensititre™ YeastOne Broth. Each strain was tested in triplicated. Positive controls (untreated) and efficiency controls performed using fluconazole (tested concentration range 256–0.25 μg/mL) were included. Efficiency controls were performed according to International guide line CLSI (Clinical and Laboratory Standard Institute). Plates were incubated at 37 °C in aerobiosis and after 24 h, MIC values were determined by spectrophotometric reading at 450 nm (EL808, Biotek, Winooski, VT, USA). To determine MFC, 5 μL of the content of each well were seeded on Sabouraud Dextrose Agar plates, which were incubated at 37 °C for 24/48 h. The MFC was defined as the lowest concentration with the death of almost the 99.9% of the initial inoculum.

### 4.10. SEM Evaluation

The morphology of *Candida* spp. cells after treatment with CIN and CIN-MPs, at MIC and sub-MIC concentrations, was examined by scansion electron microscopy (SEM, Supra 25′, Zeiss, Germany). The samples were grown on 13 mm diameter round basic slides (Agar Scientific, Stansted, UK) in the wells of a 24-well plate, according to the protocol described above. The slides were then stabilized in a solution of a 2.5% glutaraldehyde for 15 min and dehydrated in serial dilutions (30, 50, 70, 90, 100% *v*/*v*) of ethanol for 10 min. Subsequently, the slides were air-dried and then transferred to copper disks and metallized with a 40 nm thick gold film using a sputter coater (High Resolution Sputter Coater AGB7234, UK) (19.30 g/cm^3^ and 40 mA/s) before analysis by SEM. Observations were performed at 1.5kV–14.7kV.

### 4.11. Effect of CIN and CIN-MPs on Cell Membrane Integrity

The destruction of cell membrane integrity was expressed by determination of the nucleic acid content in the fungal suspension. The fungal suspension adjusted to 5 × 10^5^ CFU/mL in Sensititre™ YeastOne Broth, were incubated on a rotary shaker with various concentrations of CIN/CIN-MPs (range concentration from 1250 µg/mL to 300 µg/mL) at 37 °C for 24 h. Positive controls were included. The supernatant, obtained by centrifugation at 4000 rpm for 10 min, was filtered with a 0.22 μm filter and the nucleic acid concentration was determined by spectrophotometric reading at 260 nm by UV-vis spectrophotometer (UV-2100, Unico Instrument Co., Ltd., Shanghai, China).

### 4.12. Statistical Analysis

The GraphPad Prism v.8 software (GraphPad Software Inc., San Diego, CA, USA) was used to perform Statistical analysis using Two-Way ANOVA test. In order to correct the multiple comparisons were used: an ordinary Dunnett’s (*p* < 0.05) multiple-comparison to study both the effectiveness of CIN and CIN-MPs on nucleic acid release and their toxicity on *G. mellonella*; a Šídák’s multiple comparisons test for the in vitro release studies; a Tukey’s multiple comparisons test to study antifungal activity. Normal distribution data were analysed using mean and standard deviation parameters. *p* values < 0.05 were considerd significant.

## 5. Conclusions

The antimicrobial properties of *Cinnamomum cassia* EO and cinnamaldehyde, its main component, are widely demonstrated. Unfortunately, the volatility and poor solubility in water make these natural compounds difficult to use in clinical practice. The use of appropriate delivery systems for the encapsulation of phytocompounds overcomes these limitations as well as having an impact on the side effects of possible therapies. Our data demonstrate that cinnamaldehyde incorporated in PLGA microparticles is stable, has optimal time release, is safe and has effective antifungal properties against clinical isolates of *C. albicans* and *C. glabrata* susceptible and resistant to common antifungal drugs.

## Figures and Tables

**Table 1:** Chemical volatile composition (percentage mean values ± SD) of CC-EO and vehiculated CIN.

N°	COMPONENT ^1^	LRI ^2^	LRI ^3^	CC-EO ^4^ (%)	CIN-MPs ^5^ (%)
1	styrene	894	898	1.0 ± 0.01	-
2	*α*-pinene	938	943	1.6 ± 0.02	-
3	benzaldehyde	940	945	2.7 ± 0.05	-
4	camphene	942	946	1.4 ± 0.04	-
5	*β*-pinene	978	986	0.5 ± 0.02	-
6	*p*-cymene	1018	1021	0.3 ± 0.01	-
7	limonene	1024	1026	0.7 ± 0.03	-
8	salicylaldehyde	1050	1057	0.3 ± 0.02	-
9	phenylethyl alcohol	1100	1102	0.7 ± 0.02	-
10	3-methylacetophenone	1158	*	0.1 ± 0.01	-
11	benzenepropanol	1163	1162	0.6 ± 0.02	-
12	borneol	1165	1163	0.5 ± 0.02	-
13	*α*-terpineol	1190	1183	0.1 ± 0.01	-
14	cis-cinnamaldehyde	1210	1215	0.8 ± 0.02	-
15	*O*-anisaldehyde	1238	1240	0.8 ± 0.02	-
16	trans-cinnamaldehyde	1270	1275	70.4 ± 1.15	100.0
17	ylangene	1374	1376	0.4 ± 0.01	-
18	*α*-copaene	1388	1392	9.8 ± 0.05	-
19	*β*-caryophyllene	1437	1440	1.8 ± 0.03	-
20	aromadendrene	1455	1460	0.5 ± 0.02	-
21	*α*-curcumene	1480	1485	0.6 ± 0.02	-
22	*γ*-muurolene	1488	1486	1.1 ± 0.04	-
23	*β*-bisabolene	1498	1495	0.7 ± 0.03	-
24	ledene	1500	1496	0.1 ± 0.01	-
25	*α*-muurolene	1505	*	0.5 ± 0.03	-
26	*δ*-cadinene	1533	1530	1.7 ± 0.02	-
27	spathulenol	1604	1601	0.2 ± 0.02	-
28	*α*-bisabolol	1680	1674	0.1 ± 0.01	-
	SUM			100.0	100.0

^1^ the components are reported according to their elution order on apolar column; ^2^ Linear Retention Indices calculated using the apolar column; ^3^ Linear Retention indices from literature; * LRI not available; CC-EO ^4^: Percentage mean values of pure CC-EO components; CIN-MPs ^5^: Percentage mean values of vehiculated CIN components; - Not detected.

**Table 2:** Cumulative percentage of released CIN in ethanol and Sensititre YeastOne broth at 1, 4 and 4 and 24 h. Analyses were performed in triplicate (*p* value < 0.0001).

Timepoints	Cumulative Percentage (% *v*/*v*) ± St. Dev.	
	In Ethanol	In Sensititre™ YeastOne Broth
1 h	67.13 ± 2.20	17.65 ± 1.75
4 h	93.347 ± 2.16	21.22 ± 2.20
24 h	99.67 ± 1.58	26.30 ± 2.13

**Table 3:** Release percentage of CIN on a growth area of 0.31 cm^2^ and 1.90 cm^2^ in 24 h at 600, 300 and 150 µg/mL of CIN-MPs. Analyses were performed in triplicate.

CIN-MPs Concentrations	Release Percentage (% *v*/*v*) ± St. Dev.	
	0.31 cm^2^	1.90 cm^2^
600 µg/mL	61.85 ± 1.44	85.66 ± 2.02
300 µg/mL	74.43 ± 1.57	92.85 ± 1.69
150 µg/mL	89.00 ± 1.75	93.00 ± 1.32

**Table 4:** MFC90 values of CIN, CIN-MPs, CC-EO and empty MPs against *C. albicans* and *C. glabrata* strains. Analyses were performed in triplicate.

		MFC90 μg/mL		
	*C. albicans* R	*C. albicans* S	*C. glabrata* R	*C. glabrata* S
CIN vehiculated	200 ± 77	400 ± 155	400 ± 155	450 ± 164
CIN	350 ± 123	500 ± 155	500 ± 155	500 ± 155
CC-EO	400 ± 173	500 ± 173	500 ± 173	500 ± 173
Empty MPs	>40,000	>40,000	>40,000	>40,000

**Table 5:** Nucleic acid (OD260nm) released from Candida strains untreated and treated with CIN and CIN-MPs at MFC90 concentrations. Analyses were performed in triplicate (*p* value < 0.0001).

Nucleic Acid Absorbance ± St. Dev.				
	*C. albicans* R	*C. albicans* S	*C. glabrata* R	*C. glabrata* S
CIN-MPs	0.54 ± 0.07	0.56 ± 0.05	0.69 ± 0.08	0.82 ± 0.03
CIN	0.75 ± 0.04	0.94 ± 0.05	0.65 ± 0.03	0.68 ± 0.07
Untreated (Control)	0.20 ± 0.01	0.25 ± 0.05	0.26 ± 0.02	0.34 ± 0.04

## Data Availability

All generated data are included in this article.
